# Expression of GNAQ, BAP1, SF3B1, and EIF1AX Proteins in the Aqueous Humor of Eyes Affected by Uveal Melanoma

**DOI:** 10.1167/iovs.65.1.15

**Published:** 2024-01-04

**Authors:** Giulia Midena, Raffaele Parrozzani, Luisa Frizziero, Graziana Esposito, Alessandra Micera, Edoardo Midena

**Affiliations:** 1 IRCCS–Fondazione Bietti, Rome, Italy; 2 Department of Ophthalmology, University of Padova, Padova, Italy

**Keywords:** uveal melanoma (UM), liquid biopsy, aqueous humor (AH), proteomic, biomarkers

## Abstract

**Purpose:**

The purpose of this study was to quantify specific aqueous humor (AH) proteins in eyes affected by posterior uveal melanoma (UM).

**Methods:**

Thirty-six eyes affected by primary UM were included. Tumor thickness and largest basal diameter were specific clinical characteristics. Tumors were staged with the American Joint Commission on Cancer Eighth Edition (AJCC) classification. During the brachytherapy (Iodine-125) surgical procedure, both the AH sample collection and the 25-gauge transscleral fine needle aspiration biopsy (FNAB) were performed. AH samples were analyzed by immunoprecipitation and SDS PAGE techniques to quantify GNAQ, BAP1, SF3B1, and EIF1AX proteins. Cytologic material underwent fluorescence in situ hybridization for chromosome 3. The AH of 36 healthy eyes was used as the control group. Cluster analysis of groups was also performed.

**Results:**

Compared with the control group, significantly higher protein levels of: GNAQ (*P* = 0.02), BAP1 (*P* = 0.01), and SF3B1 (*P* = 0.02) were detected in eyes with UM. Cluster analysis of UM group revealed 2 clusters, one showing higher expression of GNAQ and BAP1 protein and one of EIF1AX protein. Moreover, the 2 clusters corresponded with the chromosome 3 status of UM.

**Conclusions:**

Specific and selected proteins may be detected in the AH of eyes affected by UM. These findings confirm the possibilities provided by AH analysis in UM.

Uveal melanoma (UM) is the most common primary intraocular malignancy in adults. The primary tumor can be successfully treated, but, unfortunately, prevention and treatment of the metastatic diseases are still unsolved, and nearly half of the patients eventually develop fatal metastatic disease.^1^^–^^5^ Clinical, histopathologic, and genetic features have been identified as indicators of UM prognosis. Especially, almost all UM carry a driver genetic mutation in a GNA (guanine nucleotide-binding protein subunit alpha) family gene, including GNAQ, which encode guanine nucleotide-binding protein G(q) subunit alpha.^1^^–^^5^ Most UM that do not harbor a mutation in GNAQ have a mutation in its paralogue GNA11 and as these mutations are mutually exclusive analyzing one, we can consequentially define the other. Moreover, mutation in other genes linked to the Gα11/Q pathway (PLCB4 and CYSTLR2) are involved in UM pathogenesis in those tumors that do not harbor GNAQ or GNA11 mutations. Furthermore, specific genetic features associated with metastatic disease include loss of chromosome 3 and mutations in the BAP1 (BRCA-associated protein 1) and SF3B1 (encoding splicing factor 3B subunit 1A) genes. BAP1 mutations are observed in approximately half of all cases of UM and usually result in metastasis within 5 years, whereas UM with a mutation in the EIF1AX (encoding eukaryotic translation initiation factor 1A X-linked) gene infrequently metastasize.^6^^–^^9^ GNAQ gene encodes the alpha subunit of heterotrimeric G proteins, which couple seven-transmembrane domain receptors to intracellular signaling machinery. This alpha subunit is useful as a molecular switch for the G protein, which is active when bound to guanosine triphosphate (GTP) and inactive when GTP is hydrolyzed. Substitutions of arginine or glutamine residues in the alpha subunit stopped the GTPase activity and the G protein stays in a constitutively active state.^8^^,^^10^^–^^14^ BAP1, encoded by the BAP1 gene, is a ubiquitin carboxy-terminal hydrolase, an enzyme responsible for eliminating ubiquitin from protein substrates.^8^^,^^15^ BAP1 was initially shown in cell nucleus where its primary interaction was binding to the BRCA1 and enhancing its tumor suppressive activity. Subsequently, it has been documented that BAP1 acts independently as a tumor suppressor, using its deubiquitinating activity to regulate proteins involved in cell proliferation, cell cycle control, cellular differentiation, DNA damage repair, chromatin modulation, cell death, and immune response.^16^ The SF3B1 gene encodes subunit 1 of the splicing factor 3b protein complex. Splicing factor 3b, with splicing factor 3a, and an RNA unit, forms the U2 small nuclear ribonucleoproteins complex (U2 snRNP). The splicing factor 3b/3a complex binds pre-mRNA and may anchor the U2 snRNP to the pre-mRNA. Alternative splicing results in multiple transcript variants encoding different isoforms.^17^^–^^20^ EIF1AX, encoded on human chromosome X, is a small protein and a component of the 43S pre-initiation complex, which is involved in the recruitment of the small 40S ribosomal subunit to messenger RNA. EIF1AX regulates cell proliferation, but its precise functions are poorly understood and, as a consequence, the cellular mechanisms underlying its function are still unclear.^21^

Currently, all these genes are mainly sequenced on tissue samples of UM (after eye enucleation) and only very recently Im et al. demonstrated that tumor nucleic acids are present and quantifiable in the aqueous humor (AH) of UM eyes.^22^ Nevertheless, a limited number of studies have been conducted on genes transcription end-products.^23^^–^^32^ Specifically, several authors demonstrated a strong association between BAP1 protein immunostaining and BAP1 mutation status, but no studies have been conducted on other genes/proteins with an established prognostic role.^23^^–^^35^ In a previous study, we proved that the detection of UM-related molecules in AH allowed to better understand UM pathogenesis and spreading, and previously Wierenga et al. demonstrated that AH proteomic analysis revealed the presence of different prognostic UM clusters.^25^ Specific UM biomarkers, mirroring the tumor genetic status have never been identified and quantified in AH.^36^ Moreover, several authors demonstrated that proteomic analysis of AH has the potential to add new and specific information about the pathophysiology and the prognosis of ocular disorders, even at the chorioretinal level.^36^^–^^38^ Finally, proteomic studies performed on different chorioretinal diseases defined a specific correlation not only with the pathophysiology of the disease, but also with the stage of the disease itself. This represents a milestone in the liquid biopsy approach in ophthalmology.^36^

The aim of the present study was to evaluate the presence of UM specific proteins in AH in eyes affected by UM at the time of tumor treatment.

## Materials and Methods

This was a cross-sectional case-control study with prospective enrollment, performed at the Ophthalmology Department of the University of Padova - IRCCS G.B. Bietti Foundation, Oncology and Toxicology Unit. Subjects were recruited from those referred between April and December 2020. Informed consent was obtained from each subject and the Institutional Review Board approved the study protocol. The approval for the study was obtained from the local ethics committee (study N.:4828 Prot. N. 18682, February 20, 2020). Subjects were diagnosed by a senior ocular oncologist by ophthalmoscopy and A-B scan ultrasonography. Both liver enzymes and liver ultrasonography were used to evaluate the metastatic disease at baseline. Inclusion criteria were: subjects affected by UM and planned to be treated with Iodine-125 (I-125) brachytherapy according to the Collaborative Ocular Melanoma Study (COMS) guidelines (85 Gy at tumor apex with a dose rate of 0.60–1.05 Gy/h). Exclusion criteria were: any history or clinical evidence of ocular and/or systemic diseases (e.g. diabetes), any previous ocular surgery, intravitreal drug injection or laser treatment, and any significant refractive error (>6 diopters [D]). Patients affected by metastatic UM at baseline were also excluded. A control group presenting for cataract surgery was prospectively recruited during the same period, for AH sampling. Exclusion criteria for the control cases were strict: subjects with any previous or present ocular disorder, any previous ocular surgical procedure, recent (6 months) local treatment of the eye, or subjects affected by any significant systemic disease, were excluded from the study, in order to avoid confounding data. Therefore, also control eyes underwent a full ophthalmologic evaluation. Therefore, healthy controls were represented by a group of age- and gender-matched subjects, unaffected by concomitant relevant systemic or ocular disorders, which may act as confounders of biomarker quantification.

### Study Procedures

Patients planned for brachytherapy underwent AH sampling during I-125 surgical procedure, before plaque positioning, whereas healthy subjects included in the control group, matched for age, underwent AH sampling at the time of cataract surgery. Moreover, immediately before plaque positioning, fine needle aspiration biopsy (FNAB) procedure was performed in the UM group. Each enrolled subject underwent a complete ophthalmologic examination, including slit-lamp biomicroscopy and ophthalmoscopy. The location of the tumor was reported according to the main involved sector (nasal, temporal, superior, or inferior) and the tumor origin (choroid or ciliary body). Tumor thickness and largest basal diameter, measured by A- and B-scan ultrasonography (Aviso; Quantel Medical, Clermont-Ferrand, France) were also reported. Tumors were staged using the American Joint Commission on Cancer (AJCC) Eighth Edition classification.

### Aqueous Humor Sample Collection and Preparation for Analysis

Each enrolled subject underwent standard pre-operative preparation for eye surgery, including disinfection of periocular skin with povidone-iodine 10% (ESO JOD; ECOLAB, Agrate Brianza, Italia), irrigation of the conjunctival sac with povidone iodine 5% (Oftasteril, Alphaintes), washing out of the eye with balanced salt solution. AH (150–200 µL/sample) was aspirated from the anterior chamber, using a 30-gauge needle connected to an insulin syringe (1 mL). After aspiration, AH was collected by a second operator in a single microfuge containing 10 µL of a cocktail of protease inhibitors (Pierce Biotechnology, Rockford, IL, USA). Coded microvials were quickly stored at −80°C. The total protein content was quantified in 3 µL/sample with a digital spectrophotometer (NanoDrop; Thermo Fisher Scientific Inc., Waltham, MA, USA) and protein concentrations were calculated by means of the linearized standard curve (bovine serum albumin) using the A280 application. AH samples where than sonicated (VibraCell; Sonics, Newton, CT, USA) and clear supernatants were provided by centrifugation (13,000 rpm/7 minutes). The amount of total proteins was used for normalization purpose, because multiparametric assays were performed.

### Immunoprecipitation, SDS PAGE, and Immunoblotting

Not-pooled samples were used for these studies. Targets were analyzed according to the direct immunoprecipitation (IP) technique coupled to Western blotting analysis. The capture antibodies specific for GNAQ (SAB2501681; Sigma), BAP1 (HPA028815; Sigma), EIF1AX (1SAB2700577; Sigma), and SF3B1 (SAB2108710; Sigma) were pre-incubated with Pure Proteome Protein G Magnetic beads (15 µL; Millipore, Burlington, MA, USA) and immobilized with a magnet, to generate the antibody-beads complex. The beads-bound antibodies were then added to the normalized samples (30 µg total protein/sample) and after 2 hours of incubation, the specific bounds were separated, washed, and eluted in denaturing Loading Buffer. All steps were performed under orbital shaking (Certomat II, Sartorius AG). Loading Buffer and samples were preheated at 90°C for 10 minutes and loaded on 4% to 12% precasted SDS-PAGE gels (Bio-Rad Laboratories Inc., Hercules, CA, USA) and electrophoresis was performed in a MiniProtean3 apparatus (Bio-Rad) under reducing conditions (120 V/frontline). Electrophoresed bands were transferred to 0.22 µm membranes (Hybond; GE Healthcare, Buckinghamshire, UK) at 12 V/40 minutes in a semidry Trans-Blotting apparatus (Bio-Rad). Membranes were stained with the high-sensible Sypro Ruby protein blot stainer to verify the presence of specific bands (Invitrogen, Waltham, MA, USA), according to a standard procedure. Immunoblotting with specific detection antibodies and chemiluminescent developing were performed to visualize and acquire the target of interest. The analysis of Integrated Density (IntDen) was performed for each band, using the free available ImageJ software (Image J version 1.43; National Institutes of Health [NIH] http://rsb.info.nih.gov/ij/). Images were saved as 8-bit TIFF files and data were exported for figure assembly using the Adobe Photoshop (2022) version 22.0.0 software (Adobe Systems Inc., San Jose, CA, USA).

### Fine Needle Aspiration Biopsy

Fine needle aspiration biopsy (FNAB) procedure was performed using a 25-gauge spinal needle connected to a 10 cc syringe by a hollow tube. The needle was inserted into the tumor through a 300 µm scleral incision (to avoid excessive pressure when penetrating the eye). A double-pass sampling was performed. The scleral incision was sutured and the plaque immediately placed over the tumor base. Tumor specimens obtained by FNAB were collected in culture medium RPMI 1640 (Euroclone Life Science, Pero-MI, Italy).

### Cytogenetic Analysis

The sampled material underwent fluorescence in situ hybridization (FISH). After sedimentation, the material was enzymatically digested with collagenase II (Worthington Biochemical Corporation, Lakewood, NJ, USA) 1400 U/mL at 37°C for 2 hours. The suspension was washed in RPMI 1640 and used to prepared cytospins. Slides were fixed with a cytologic fixative (Bio- Fix; Bio-Optica, Milano, Italy), and stored at –20°C. FISH analysis was performed with a centromeric probe for chromosome 3 labeled with SpectrumOrange and centromeric probe for chromosome 10 labeled with Spectrum Green (Abbott-Vysis, Downers Grove, IL, USA) following the manufacturer's procedure. Slide and probe were codenatured in Hybrite’ (Vysis) at 75°C for 5′ and hybridized in a humid chamber overnight at 42°C. Post-hybridization washes were made at 73°C in 0.4 × SSC/0.3% NP-40 for 2′ and at room temperature in 2 × SSC/0, 1% NP-40 for 1′. Slides were air dried and mounted with a Vectashield‚ mounting medium with DAPI (Vector Laboratories, Burlingame, CA, USA). Microscope analysis was carried out with a fluorescent microscope (Zeiss Axioplan fluorescent microscope, Jena, Germany) equipped with a cooled charge-coupled device (CCD) camera (Hamamatsu, Hamamatsu City, Japan) and appropriate single band and triple band filters. Images were analyzed using CRO- MOFISH software (Amplimedical, Assago-MI, Italy). At least 100 cells were evaluated for each case; loss of chromosome 3 was reported when more than 15% of cells showed a single signal for chromosome 3.

### Statistical Analysis

Data are presented as the mean ± standard deviation, and the normality of the distribution was assessed by the Shapiro–Wilk test. The comparison of AH proteins’ expression in patients with UM and in controls and was made, for each protein, by means of Wilcoxon-Mann-Whitney test with Bonferroni correction of a post hoc significance level. Linear regression was applied to see whether clinical and genetic characteristics correlated with each protein level. Protein expression cutoff values were investigated. Area under the receiver operating characteristic (ROC) curve was computed and its significance was tested by Mann–Whitney test. Cutoff values were identified according to various criteria (distance to corner, sensitivity-specificity difference, and Youden index). Graduation of protein expression in the UM group was performed through quartiles of the sampling distribution. Finally, hierarchical cluster analysis (agglomerative procedure) was used to organize proteins and cases, respectively, and the Wilcoxon-Mann-Whitney test was applied to compare the clusters. Data were analyzed using SAS statistical software (SAS 9.2; SAS Institute, Cary, NC, USA). A value of *P* < 0.05 was considered statistically significant.

## Results

Thirty-six subjects with UM (36 eyes) and 36 controls (36 eyes) were included. The mean age at study inclusion was 67.5 ± 12.5 and 62.7 ± 18.4, respectively for UM subjects and healthy controls (*P* = 0.32). There was no significant difference in gender (*P* = 0.22) between the two study groups. In the UM group, 30 subjects (83%) were affected by choroidal tumor and 6 patients (17%) by ciliary body tumor. According to the AJCC Eighth Edition classification, tumor size categories were T4 in 9 eyes (25%), T3 in 17 eyes (47%), and T2 in 10 eyes (28%). The mean tumor thickness was 8.6 ± 2.6 mm and largest basal diameter 16.1 ± 2.4 mm. No complications after AH sampling and FNAB were reported in both groups.

Transscleral FNAB yielded enough material for FISH analysis in all the 36 cases (100%). Monosomy 3 was detected in 20 cases (56%) and disomy 3 in the remaining 16 cases (44%). Among the 20 monosomy 3 tumors, the mean percentage of monosomic cells in each sample was 90% ± 9.3% (range = 74–100%).

**Figure 1.**
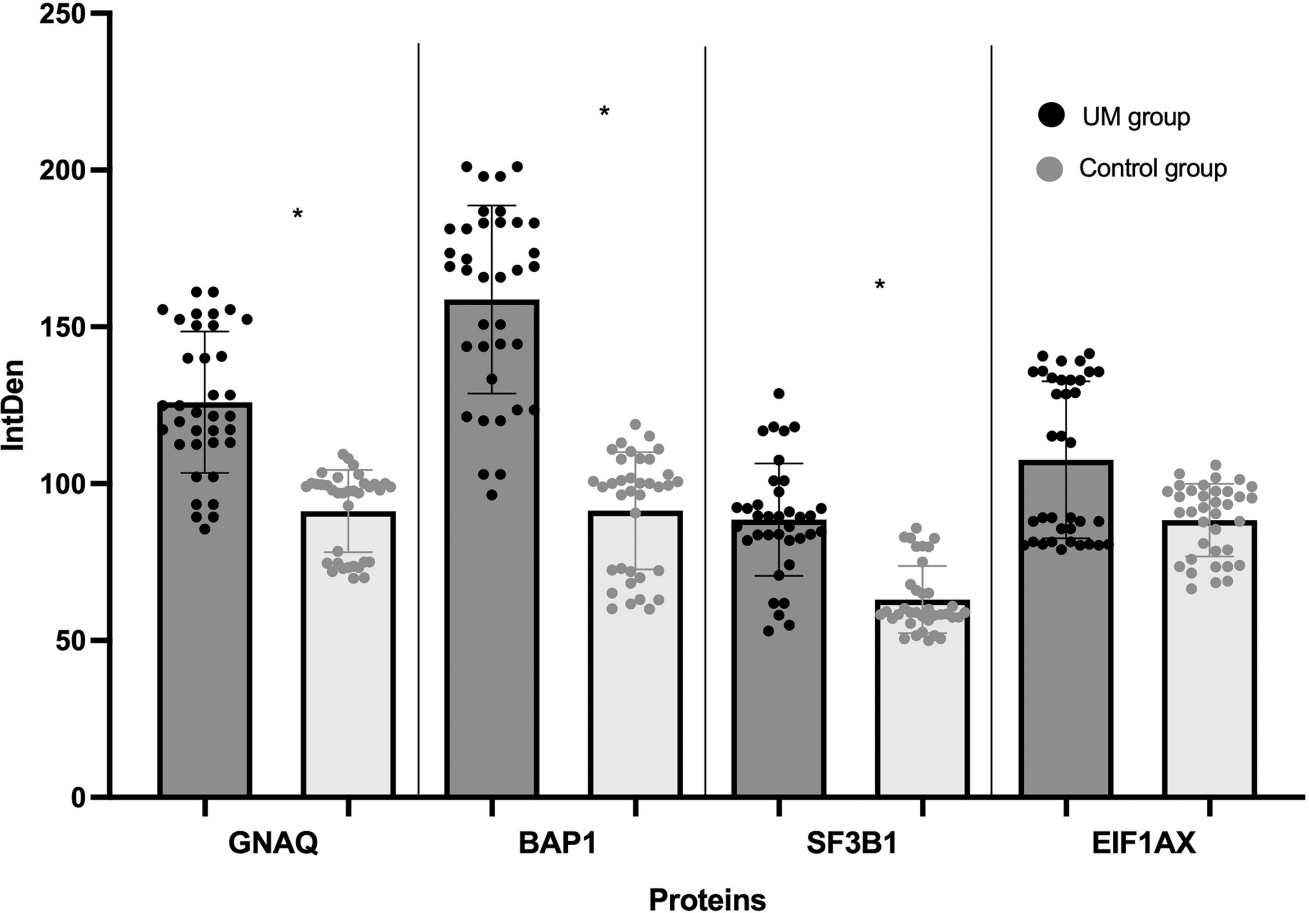
Representation of immunoprecipitation and SDS PAGE analysis of aqueous humor dosed proteins in uveal melanoma eyes (study group) versus cataract eyes (control group). Expression levels (integrated density values) are given in arbitrary units. * = proteins showing a statistically significant difference (*P* < 0.05) between study and control group. UM, uveal melanoma; IntDen, integrated density.

GNAQ, BAP1, SF3B1, and EIF1AX levels in the AH samples are shown in Figure 1. Compared to the control group, significant higher levels of GNAQ (*P* = 0.02), BAP1 (*P* = 0.01), and SF3B1 (*P* = 0.02) were detected in eyes with UM.

No statistically significant correlations between clinical features (age, largest basal diameter [LBD], tumor height, tumor stage) and proteins expression were observed. Moreover, no statistically significant correlations among GNAQ, BAP1, SF3B1, and EIF1AX protein expression and chromosome 3 status were reported.

Furthermore, mean values of proteins expression were also analyzed in order to find cutoff values to identify different classes of protein expression, as shown in Table 1.

**Table 1.** Cutoff Values of Protein Expression in Uveal Melanoma

	Expression Levels		
Proteins	Low	Medium	High
GNAQ	<112.8	112.8–145.5	>145.5
BAP1	<128.4	128.4–182.2	>182.2
EIF1AX	<86.8	86.8-135.7	>135.7
SF3B1	<81.9	81.9-93.3	>93.3

**Figure 2.**
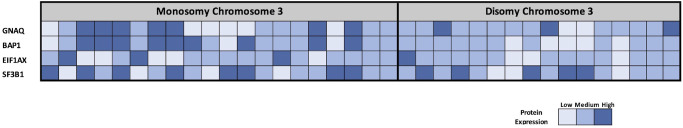
Heatmap showing expression of GNAQ, BAP1, EIF1AX, and SF3B1 for each UM subject, divided according to chromosome 3 status.

The protein expression levels for each UM subject were recorded and expressed with the information regarding chromosome 3 status, as shown in Figure 2.

**Figure 3.**
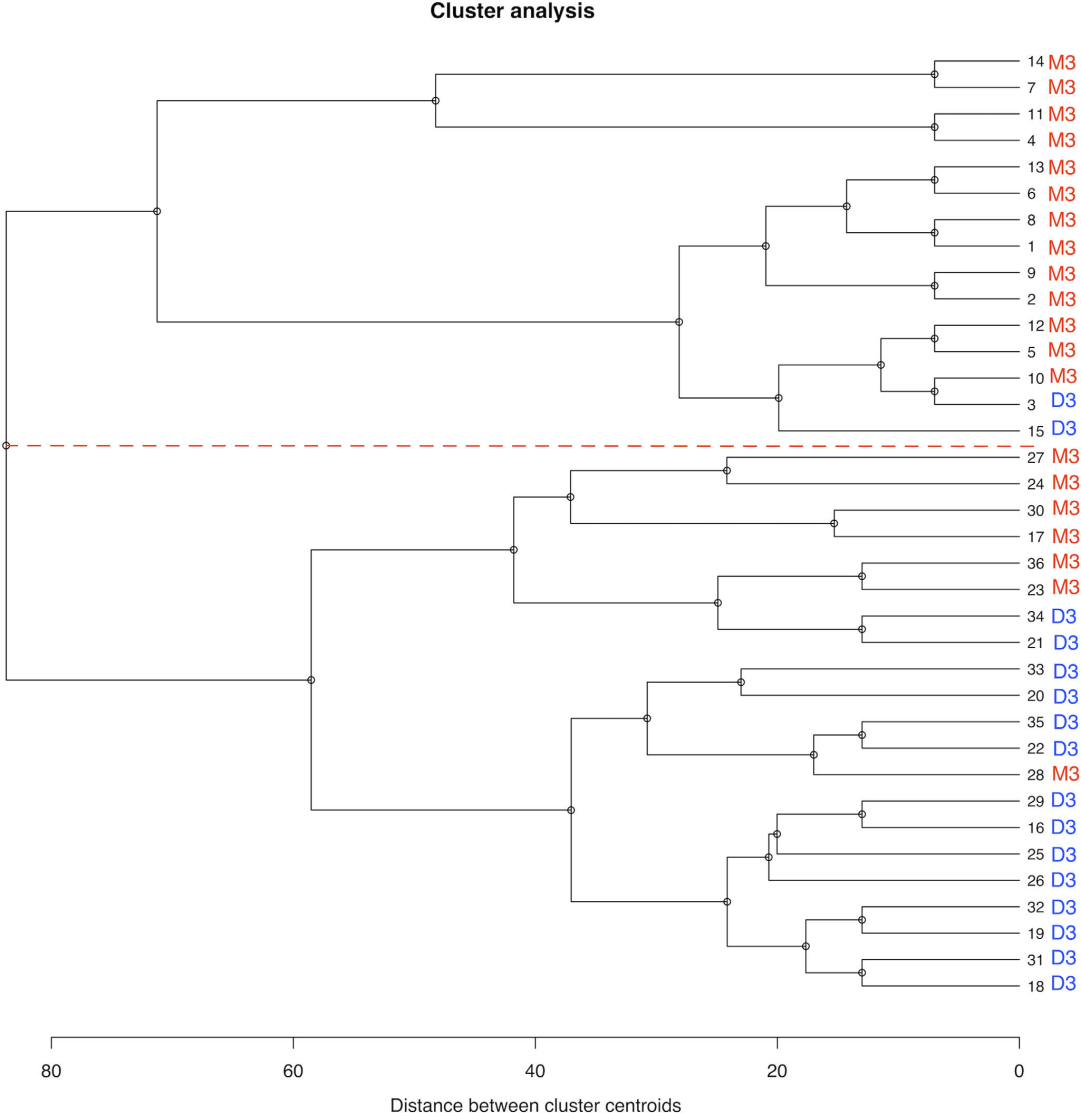
Dendrogram of hierarchical cluster analysis (agglomerative procedure) of the whole UM group. The *red dotted line* separates cluster 1 (dendrogram *upper section*) from cluster 2 (dendrogram *lower section*). Cluster variables are: GNAQ, BAP1, SF3B1, and EIF1AX protein absolute levels. M3, monosomy chromosome 3; D3, disomy chromosome 3.

**Figure 4.**
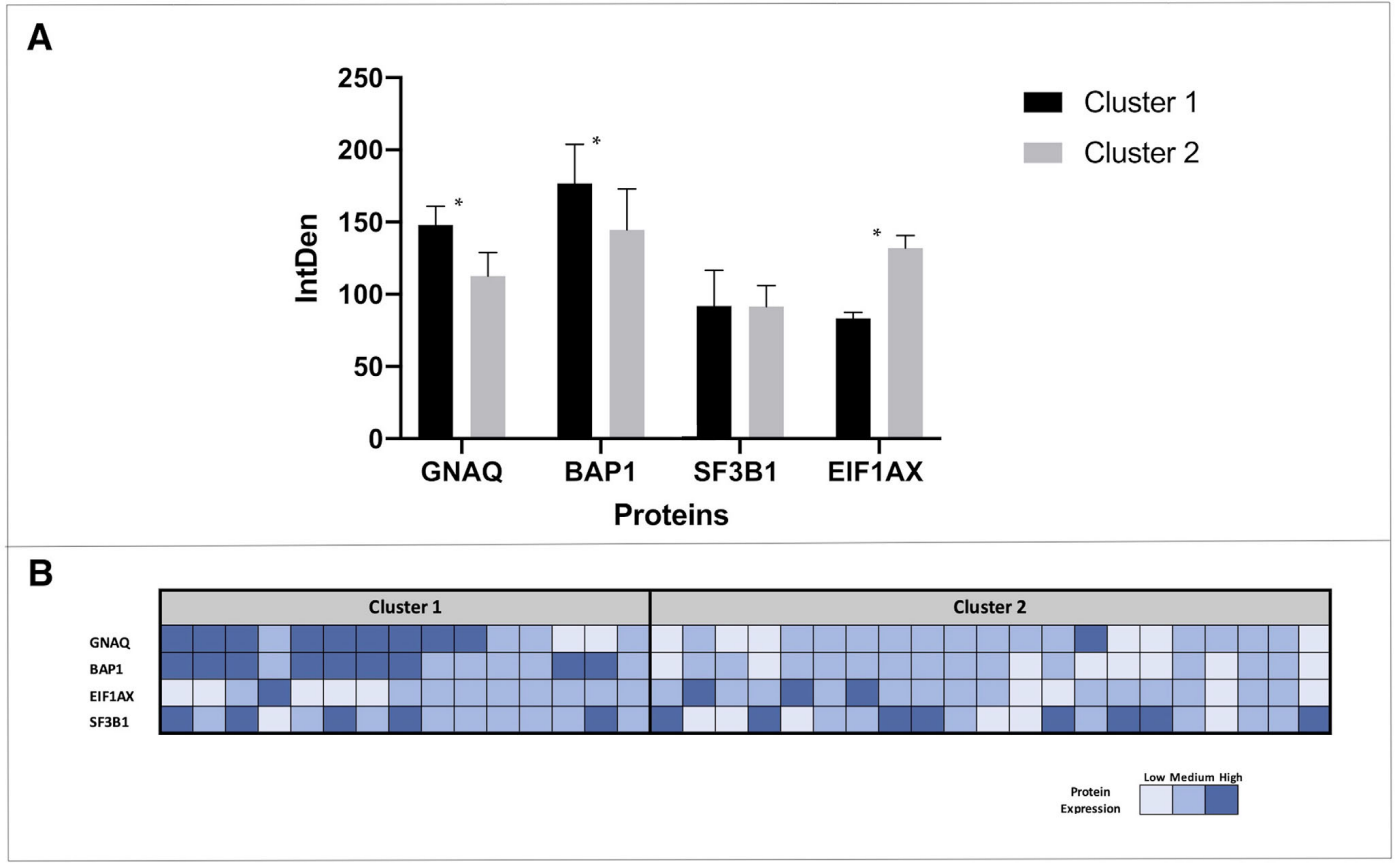
Representation of immunoprecipitation and SDS PAGE analysis of aqueous humor proteins dosed by clusters. Expression levels (integrated density values) are given in arbitrary units (**A**). Data are shown as mean ± SD. * = proteins showing a statistically significant difference (*P* < 0.05) between clusters. Heatmap showing expression of GNAQ, BAP1, EIF1AX, and SF3B1 for each UM subject, divided according to cluster (**B**).

Furthermore, hierarchical cluster analysis (agglomerative procedure) of the UM group revealed two clusters, as shown in Figure 3. In particular, one consisting of 15 cases showing higher expression of GNAQ and BAP1 and low presence of EIF1AX; another consisting of 21 cases with higher expression of EIF1AX and lower expression of GNAQ and BAP1, as shown in Figure 4. The *P* values for the linkage of all the selected proteins were < 0.05. The 2 clusters differed significantly in chromosome 3 status (*P* = 0.02), with the cluster with more expression of GNAQ and BAP1 showing monosomy 3. The features of each cluster are summarized in Table 2. Cluster analysis has been performed also for the control group and it showed no statistically significant evidence for clustering, without overlaps with cluster 2.

**Table 2.** Characteristics of Clusters

	Cluster 1	Cluster 2	P Value
Patients, no.	15	21	
Tumor size category: no. (%)			
T1	0 (0)	0 (0)	NA
T2	4 (27)	6 (29)	0.34
T3	7 (47)	10 (48)	0.23
T4	4 (27)	5 (24)	0.17
Tumor location (%)			
Choroid	13 (0)	17 (0)	0.37
Ciliary body	2 (0)	4 (0)	0.48
Chromosome 3 status: no. (%)			
Monosomy 3	13 (87)	7 (33)	**0.02**
Disomy 3	2 (13)	14 (67)	

no., number; y, years; SD, standard deviation.

Significant *P* values are in bold.

Finally, the protein expression levels for each UM subject were recorded and expressed with the information regarding cluster, as shown in Figure 4.

## Discussion

Several clinical, histopathological, and genetic markers have been identified to define UM features and prognosis. Classically, the TNM staging, the cell type (epithelioid cells), and the chromosome 3 status (monosomy 3) have been identified as unfavorable prognostic factors. Currently, FISH is a well-established genetic method for UM analysis and it uses oligonucleotide probes to detect chromosomal abnormalities. Moreover, it shows a good concordance with other techniques, as multiplex ligation-dependent probe amplification (MLPA) and single nucleotide polymorphism (SNP)-array.^39^ In addition, it has also been established that the tumor microenvironment and tumor genetic characteristics play a leading role in UM. The tumor microenvironment is the environment surrounding the tumor, and it includes: extracellular matrix, blood vessels, inflammatory/immune cells, and signaling molecules.^1^^,^^8^ The tumor is closely related to the microenvironment and therefore it reflects the nature of the tumor itself. It is reported also for UM that the inflammatory/immune cells infiltration is related to the prognosis. The inflammatory phenotype of UM, characterized by high infiltration of lymphocytes and macrophages is associated to a poor outcome. Moreover, recent findings reported that the microenvironment influences the expression of inhibitory immune checkpoints.^8^^,^^29^^,^^39^ Finally, concerning the recent genetic landscape, mutations in GNAQ are frequently observed in primary UM. BAP1 and SF3B1 mutations are found most frequently in metastatic disease, whereas EIF1AX gene mutation is related to a favorable prognosis.^1^^,^^8^^,^^34^ Therefore, UM is characterized by chromosomal anomalies and mutated genes.^7^^–^^9^ Activating GNAQ mutations were found in about 57% of the UM, and in addition are reported mutations in one of the three secondary driver genes. Usually 44% of UM showed a BAP1 mutation, 26% a mutation in SF3B1, and 18% a mutation in EIF1AX.^7^^–^^9^ Several studies focused on these genetic alterations, mainly analyzing UM histologic samples and recently focusing on circulating biological materials.^36^^,^^39^^–^^44^ In particular, studies on UM have identified aqueous and vitreous humor as sources of circulating tumor DNA, but no studies have been specifically conducted on GNAQ, BAP1, SF3B1, and EIF1AX related proteins.^45^^–^^48^ The correlation between proteins’ concentration in vitreous and AH has already been reported, confirming the value of aqueous sampling as a safe and less invasive procedure compared to the vitreous one.^46^^,^^49^ Because of the identification of aqueous flare in UM eyes, the relevance of soluble factors interactions in UM microenvironment has been suggested.^36^^,^^46^^–^^48^ Moreover, in a previous study, we have identified in the AH of UM subjects several growth factors and inflammatory cytokines related to the tumor itself, that characterized its microenvironment.^36^ Therefore, proteomic analysis of AH in UM represents a safe and effective liquid biopsy approach in ocular oncology. In particular, UM is still a partially unknown tumor, indeed a better understanding of the complex interaction among genetic factors, molecular signaling, and targets will also help in discovering new personalized and targeted systemic therapies.^1^^,^^8^ Therefore, the identification of AH proteins (with a direct analysis) may help to recognize their potential role in pathogenesis/prognosis. We focused the present study on the identification, never performed before, of GNAQ, BAP1, SF3B1, and EIF1AX proteins in AH.

The GNAQ gene mutations result in an overactive protein, which leads to an excessive signaling, that contributes to cells overgrowth and tumor formation. Moreover, the Catalogue of Somatic Mutations in Cancer (COSMIC), showed that there is a high prevalence of GNAQ activating mutations in several tumors, including: UM, cutaneous melanoma, and colon adenocarcinoma.^50^ Furthermore, Van Raamsdonk et al. demonstrated precisely in UM cell lines that knockdown of GNAQ resulted in a decreased growth and an increased apoptosis.^14^ In our cohort, we have found in AH a significantly higher concentration of GNAQ protein in the UM group compared to the control group, therefore its overexpression in AH may reflect the hyperactive state of the protein in the tumoral tissue, which is essential for activating the tumor growth. Fortunately, even if GNAQ is essential to promote tumorigenesis, it is insufficient to induce the complete malignant transformation and in particular the aggressiveness of UM is determined by secondary driver mutations. Unexpectedly, the significant difference in GNAQ protein levels between clusters 1 and 2 may be due to the fact that GNAQ is the most common driver mutation, but not the only one. Therefore, UMs in cluster 2 probably have a greater variability in driver alterations, which are usually mutually exclusive. Regarding BAP1, several studies have demonstrated that it is usually lost or inactivated in many tumors, including UM.^51^^–^^58^ BAP1 inactivation is established to be the driving force for the development of metastasis. Moreover, BAP1 gene expression correlated with the findings of the BAP1 immunohistochemistry of the tumoral tissue.^26^^,^^27^^,^^53^^,^^56^^–^^58^ In particular, wild type BAP1 was located in the nucleus (required for the tumor suppressor activity), whereas mutant BAP1 proteins showed impaired nuclear localization and an increased cytoplasmic appearance.^34^ Our data demonstrated the overexpression of BAP1 in AH in the UM group and this may reflect the increased cytoplasmic localization of the protein. Moreover, this finding is consistent with Smit et al. that stated how some BAP1-mutated/ immunohistochemically BAP1-negative UM still show expression of BAP1, suggesting that negative nuclear staining for BAP1 may be due to an unexplained different mechanism.^8^ Furthermore, the role of cytoplasmic BAP1 in the metastasis of UM has been questioned, because a correlation between disease-free survival of subjects with UM and the cytoplasmic expression of BAP1 was not observed.^8^ Unfortunately, our data do not provide useful information on the functional state of the protein itself and further studies are needed. Moreover, another peculiar mechanism, which may explain the overexpression of BAP1, may be due to the aggressiveness of BAP1 mutated UM on surrounding cells, which rapidly die releasing proteins into the microenvironment. Interestingly, in our study, the cluster with hyperexpression of GNAQ and BAP1 showed a statistically significant association with monosomy 3. This may be due to the huge inflammatory response, strongly associated with chromosome 3 monosomy. Indeed, the inflammatory phenotype, characterized by the increase of macrophages, lymphocytes, and by the overexpression of HLA classes I and II, is associated with a worse prognosis.^23^^,^^25^^,^^46^ Moreover, this inflammatory phenotype causes the recruitment/release of more inflammatory cells in AH, which also express BAP1 and GNAQ proteins. The association between BAP1 and monosomy 3 found in cluster 2 emphasizes the importance of the association of BAP1 alterations with metastatic risk in UM subjects with complete or partial loss of 1 copy of chromosome 3. Mutations in SF3B1 have been seen in advanced chronic lymphocytic leukemia, myelodysplastic syndromes and breast cancer.^59^^–^^61^ Recently, it has been demonstrated that SF3B1 mutations are involved in UM and associated with a poor prognosis.^8^ In our cohort, we have found, in AH, a significantly higher concentration of SF3B1. EIF1AX mutations have been reported in several tumors and these mutations are assumed to result in increased or altered protein function.^50^ Mutations have been recurrently seen associated to cases of UM without monosomy 3.^8^ This is consistent with the results of our clustering analysis, in which cluster 2 showed EIF1AX hyperexpression and a statistically significant association with disomy 3. Therefore, these data confirmed that EIF1AX-mutant tumors showed a low-risk form of the disease. Probably due to the small incidence of this mutation, even if the concentration of EIF1AX protein was increased in the UM group, it did not reach a statistically significance. The identification of protein expression cutoff values identified three degrees of protein expression: low, medium, and high. The heatmap displaying expression of GNAQ, BAP1, EIF1AX, and SF3B1 for each UM subject, divided according to chromosome 3 status, clearly demonstrates that monosomy 3 UM are mainly characterized by high and medium levels of BAP1 protein expression, whereas in disomy 3 UM the levels of EIF1AX are mainly high and medium. These data, although a statistical significance was not reached, paved the way to define the UM prognostic risk on the basis of proteins expression levels. The lack of correlation among GNAQ, BAP1, EIF1AX, and SF3B1 proteins and tumor genetic (monosomy and disomy chromosome 3) and clinical characteristics (especially tumor thickness and LBD) may be due to the independence of genetics/proteomics from tumor size, as demonstrated for small UM with the intrinsic ability to metastasize, or to the limited sample size.^1^^,^^8^^,^^22^^,^^36^ Nevertheless, the heatmap displaying expression of the studied proteins for each UM subjects, according to the cluster, demonstrated the statistically significant association between monosomy 3 and in particular the high level of BAP1 expression. As mentioned above, this is probably due to both the cytoplasmatic localization of BAP1 and the release of BAP1 from aggressive UM surrounding cells.

Despite the results of our analysis, some limitations of the current study should be noticed as well. The small sample size of the study requires confirmation in a larger series of patients. Further studies, may define the correlation between BAP1 status determined through immunohistochemistry of histologic samples and AH proteomic profile, to better support the liquid biopsy approach.^62^ In the near future, it will be also interesting to compare the proteomic and genomic study using AH only, fully applying the concept of liquid biopsy, to define more precise correlation between proteins and mutational status. Moreover, additional studies, aimed to compare single gene mutations and proteins expressions in AH, are ongoing and they include also other driver mutations: GNA11, PLCB4, and CYSTLR2. Finally, data regarding the correlation between metastatic disease and protein expression in the AH of UM are of utmost importance and they deserve a more in-depth analysis, which goes beyond the main purpose of this study.

UM genetics allows to define in details the pathophysiology and prognosis of this tumor. Discoveries on the role of GNAQ, BAP1, EIF1AX, and SF3B1 are revolutionizing ocular oncology of UM. Unfortunately, genetic studies often require invasive and/or expensive techniques, whereas proteomic analysis of the AH, with the identification of cutoff values and consequently of protein expression levels, allows to obtain information on GNAQ, BAP1, EIF1AX, and SF3B1 in a relatively simple and precise way.

In conclusion, the future management of UM will be strictly linked to the concept of precision medicine, requiring a tailored approach to patients. The findings of this study not only confirm the possibilities offered by AH analysis in eyes harboring a UM, but strengthen that AH evaluation may represent the liquid biopsy approach in UM diagnosis, prognosis, and follow-up. Therefore, a simple and safe AH proteomic analysis allows us to define the expression of a huge number of proteins, including those with a prognostic role: this will allow us to guide the prognosis, even in cases where it is not possible to carry out a direct tumor sample. Moreover, the possibility of carrying out serial sampling of AH during follow-up will allow us to expand knowledge regarding the pathophysiological mechanisms of UM in response to conservative treatment.
